# Tolvaptan Add-on Therapy to Overcome Loop Diuretic Resistance in Acute Heart Failure With Renal Dysfunction (DR-AHF): Design and Rationale

**DOI:** 10.3389/fcvm.2021.783181

**Published:** 2022-01-27

**Authors:** Nhat Giang Minh, Hai Nguyen Hoang, Daichi Maeda, Yuya Matsue

**Affiliations:** ^1^Department of Cardiology, Nhan Dan Gia Dinh Hospital, Ho Chi Minh City, Vietnam; ^2^Department of Cardiovascular Medicine, Juntendo University Graduate School of Medicine, Tokyo, Japan; ^3^Cardiovascular Respiratory Sleep Medicine, Juntendo University Graduate School of Medicine, Tokyo, Japan

**Keywords:** acute heart failure, worsening renal function (WRF), loop diuretic resistance, vasopressin-2 receptor antagonist, diuretic combination therapy

## Abstract

**Background:**

Diuretic Resistance in Acute Heart Failure (DR-AHF) was designed to demonstrate the effectiveness of the early tolvaptan (a vasopressin-2 receptor antagonist) add-on therapy in patients with AHF with renal dysfunction and to provide clinical evidence of loop diuretic resistance.

**Methods and Results:**

This single-centered, open-labeled, randomized, and controlled trial enrolled 128 patients hospitalized with AHF, as participants. These patients with a wet-warm phenotype, whose estimated glomerular filtration rates are of ≥15 ml/min/1.73 m^2^ and ≤ 60 ml/min/1.73 m^2^, with a cumulative urine output of <300 ml 2 h after the first dose of intravenous furosemide, will be randomly assigned 1:1 to receive standard care with an uptitrating intravenous furosemide alone, or a combination therapy with 15 mg of tolvaptan administered once daily for 2 days. The standard furosemide treatment will follow the latest position statements of the Heart Failure Association. The primary endpoint is the cumulative urine output at 48 h. The key secondary endpoints include the improvement of fractional excretion of sodium at 6 h, the total dose of furosemide, and the incidence of worsening renal function (WRF) at 48 h.

**Conclusions:**

Although the combination of diuretic treatment has recently gained more attention due to its physiologically synergistic action, its advantages may be outweighed by the substantial risk of electrolyte disturbances and severe WRF. Further, there is no consensus on the time point for early starting of add-on therapy and for the preferred diuretic combination.

**Trial registration:**

NCT04331132.

## Introduction

Acute heart failure (AHF) represents a global burden, and more than 90% of patients with AHF experience congestion at admission, for which diuretics are the cornerstone therapy to achieve a euvolemic state (1). However, some complications, including renal dysfunction, urge us to increase the dose of diuretics to overcome diuretic resistance. High-dose loop diuretics may lead to serum electrolyte disturbances, especially hyponatremia, hypokalemia, arrhythmias, and more severe renal dysfunction (2). In the Diuretic Optimization Strategies Evaluation (DOSE) study, although the treatment with high-dose loop diuretics was not shown to be associated with adverse outcomes compared to a non-high-dose therapy, a more optimal decongestive therapy has not been developed despite the unacceptably high mortality rate (3). It is well known that the diuretic response is gradually self-limited after consecutive days of loop diuretic therapy (4). Therefore, timely modification of diuretic treatments according to the early signs of diuretic resistance, including the cumulative urine output within hours following intravenous loop diuretic administration, or combination therapy, was recommended to encourage decongestion (5).

Tolvaptan, a competitive vasopressin V2-receptor antagonist, is an oral diuretic that acts on the distal portion of the nephron. It inhibits the interaction between the antidiuretic hormone arginine vasopressin and the V2 receptor, thereby preventing the activation of the aquaporin channel systems and the consequent water reabsorption, leading to the net result of free water excretion (6). Evidence from clinical trials showed that tolvaptan, in combination with loop diuretics, is well tolerated in patients with congestive heart failure. It also ameliorates symptoms of congestion, increases sodium concentration in patients with hyponatremia, reduces loop diuretic dose, and lowers the risk of hypokalemia (7, 8). However, these findings are inconsistent among studies. Although both the EVEREST (Efficacy of Vasopressin Antagonism in heart failure) (9) and the AQUAMARINE (answering questions on tolvaptan's efficacy in patients with acute decompensated heart failure and renal failure) (10) trials confirmed the benefit of adding tolvaptan to a standard loop diuretic regimen for congestive heart failure symptoms using the Likert scale, TACTICS-HF (Targeting Acute Congestion with Tolvaptan in Congestive Heart Failure) (11) could not reaffirm these findings. Similar to decongestion efficacy, the combination of diuretic regimen including tolvaptan did not reduce the worsening renal function (WRF) rate in TACTICS-HF (11) and AQUAMARINE (10).

Moreover, it should be acknowledged that these studies did not focus on patients with diuretic resistance. Numerous studies have demonstrated that those who do not respond to intravenous loop diuretics are strongly associated with high mortality (12–14). Currently, there is no approved strategy for treating this high-risk subgroup.

Based on these considerations, diuretic resistance AHF (DR-AHF) was designed to determine whether the addition of tolvaptan to a modern diuretic strategy would result in a more optimal decongesting efficacy in AHF patients with diuretic resistance.

## Methods and Analysis

Diuretic resistance in acute heart failure DR-AHF is a single-centered, open-labeled, randomized, and controlled trial. This trial was approved by the independent ethics committee and the institutional review board at Nhan Dan Gia Dinh Hospital (registration: NCT04331132). The trial will be conducted per the principles of the Declaration of Helsinki and the International Conference on Harmonization (ICH) Good Clinical Practice guidelines. Written informed consent was obtained from participants before enrollment in the study.

### Study Population

To participate in DR-AHF, patients must be ≥20 years of age with wet-warm-phenotype AHF, whose estimated glomerular filtration rates (EGFRs) at admission are ≥15 and ≤ 60 ml/min/1.73 m^2^, and with a cumulative urine output of <300 ml, 2 h after the first dose of intravenous furosemide. The inclusion and exclusion criteria are listed in Table 1.

**Table 1:** Inclusion and exclusion criteria for the diuretic resistance in acute heart failure (DR-AHF) trial.

**Inclusion criteria**
1. Aged 20–75 years 2. Admitted to hospital with a primary diagnosis of acute heart failure with wet-warm phenotype^*^ 3. Cumulative urine volume output <300 mL within 2 h after the first dose of intravenous furosemide 4. eGFR at admission 15–60 mL/min/1.73 m^2^
**Exclusion criteria**
1. Acute coronary syndrome 2. Anuria in the first 2 h after admission 3. Sepsis 4. Consciousness impairment 5. Pregnant or breastfeeding women 6. Severe valvular heart diseases (severe valvular stenosis or regurgitation) 7. Admission sodium level >140 mEq/L 8. Serum total bilirubin > 3mg/dL 9. Serum potassium >5.5 mmol/L 10. Allergy or contraindication to tolvaptan 11. Emergency indication for hemodialysis 12. Cardiogenic shock or mechanical circulation support

* *The wet-warm phenotype is defined as a patient with ≥2 clinical signs of congestion (bibasilar rales, bilateral ankle edema, ascites, jugular venous distension) and N-terminal pro B-type natriuretic peptide (NT-proBNP) at admission ≥900 pg/ml*.

### Study Flow and Loop Diuretic Strategy

**Figure 1:**
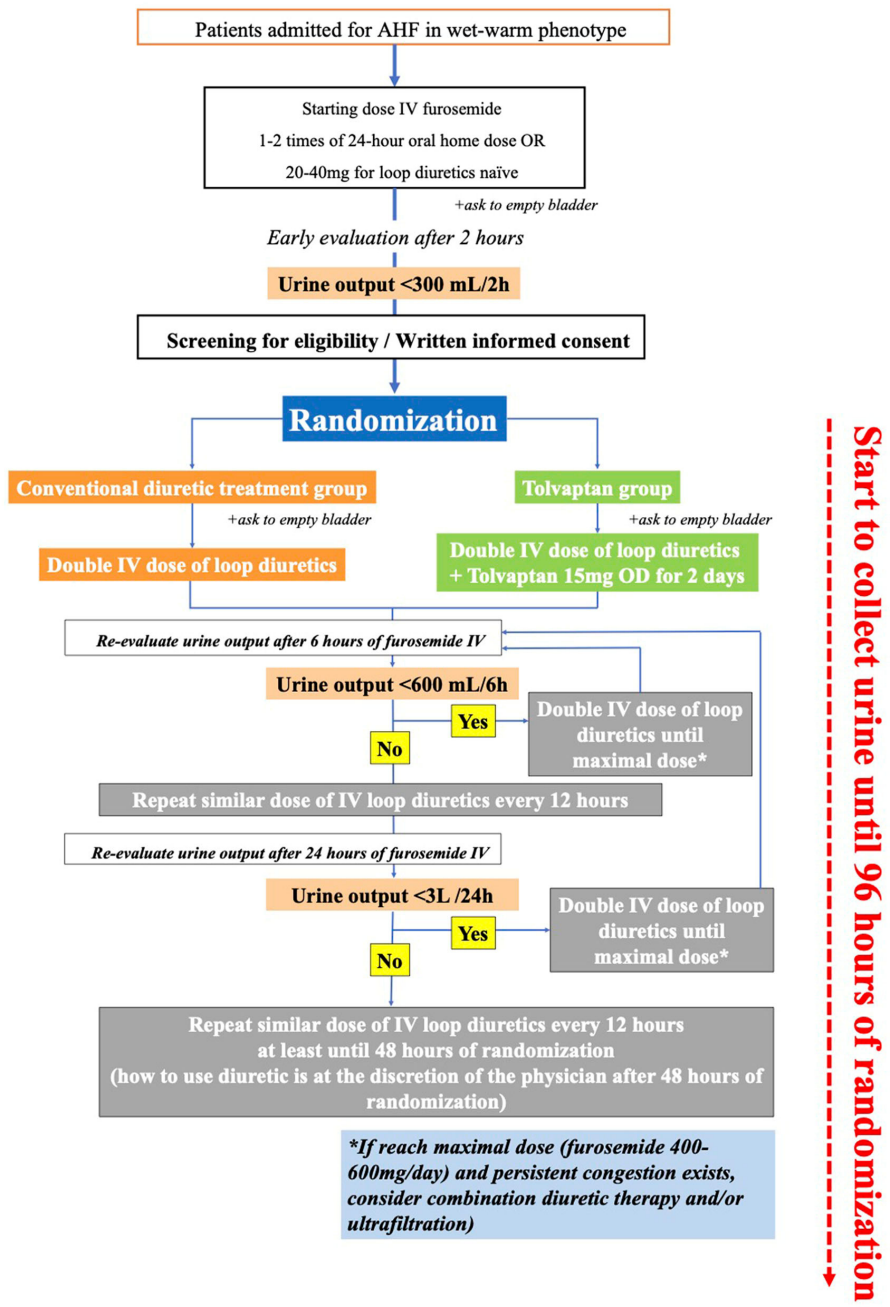
Study protocol. At 24 h, study participants will be evaluated by the same investigator (Hai H. N., Hoa K. T., Anh H. N., Thao T. N., or Tin T. H. ). If the investigator determines that the patient is absolutely hypovolemic and symptomatic, the study protocol will be ended.

On admission, patients with AHF with wet-warm phenotype will be given a starting dose of intravenous furosemide that is one to two times the 24-h oral furosemide dose prescribed before the admission home dose or 20–40 mg for treatment-naive patients with loop diuretic. Patients will be asked to immediately empty their urinary bladder before the first dose of intravenous furosemide. If eligible for inclusion in the study, at 2 h after the first intravenous furosemide, they will be randomly assigned 1:1 to receive a standard care with intravenous furosemide alone or a combination therapy with 15 mg of tolvaptan, once daily for 2 days. In both groups, the loop diuretic strategy strictly follows the modified 2019 position statement from the Heart Failure Association of the European Society of Cardiology (ESC) (5) (Figure 1). It is impossible to alter the dose of tolvaptan during the first 48 h of this study unless an adverse event was observed. After this period, the attending physicians decide whether to continue tolvaptan therapy. The standard treatments for heart failure, including the angiotensin-converting enzyme inhibitors, angiotensin II receptor blockers, vasodilators, beta-blockers, or digoxin, as well as the change from intravenous to oral loop diuretic or loop diuretic withdrawal, are left to the attending physicians 48 h after admission.

### Measurements

Urine will be collected and monitored carefully at 6 h after randomization, and the cumulative urine output will be calculated every 24 h. Vital signs and symptom improvement assessed using a 7-point Likert scale will be recorded upon admission, and 24 and 48 h after randomization. Similarly, serum sodium, potassium, and chloride will be evaluated at admission and after 12, 24, and 48 h, respectively, while urinary creatinine and sodium will be sampled at randomization and after 6 h. After randomization, serum urea and creatinine will be re-assessed at 24 and 48 h. In addition, aspartate aminotransferase (AST), alanine aminotransferase (ALT), and N-terminal pro B-type natriuretic peptide (NT-proBNP) were evaluated upon admission and at the end of the study. A summary of the study assessment time frame is presented in Appendix 1. Senior cardiologists will perform echocardiography upon admission, and at 24 and 48 h afterward, according to the echocardiography protocol described in Appendix 2.

### Endpoints

The primary endpoint was the cumulative urine output at 48 h after randomization. The secondary endpoints include the improvement of fractional excretion of sodium at 6 h, total dose of furosemide, changes in the body weight, net fluid loss, improvement of dyspnea by the 7-point Likert scale, reduction of diastolic dysfunction parameters on echocardiography, incidence of clinically relevant WRF, and changes of NT-proBNP at 48 h. Clinically relevant WRF is defined as an increase in serum creatinine level ≥0.3 mg/dl within 48 h accompanied by the doubling of furosemide dose according to the diuretic treatment protocol. A summary of the study endpoints is shown in Appendix 3.

Safety will be assessed based on the adverse effects reported throughout the study with vital signs, physical examination, clinical laboratory tests, and 12 lead-electrocardiogram. Safety topics of special interest include severe hypernatremia (plasma sodium increase > 12 mEq/24 h), hypokalemia, and hepatic injury (elevation of AST and/or ALT > three-fold the upper limit of the normal). Adverse effects and laboratory abnormalities were compared between the two treatment groups.

### Statistical Consideration

#### Sample Size

In the REALITY-AHF dataset (*n* = 1682) (15), we identified and retrieved the records of the patients treated with an intravenous furosemide within 30 min of emergency department visits. Using the ESC Heart Failure Association definition (<150 ml urine output/h), we identified 95 patients with diuretic resistance in the first 90 min, while their urine output within 48 h was 3,896 ± 1,595 ml. Assuming that the add-on tolvaptan therapy could yield 1,000 ml more urine output at 48 h, we calculated the sample size of 128 patients with an enrollment ratio of 1:1 after adjusting for 15% dropout during the follow-up period. This sample size was intended to provide a 90% power and 0.05 type I error probability.

#### Randomization

The study is an open-label randomized trial, and randomization will be performed *via* a web portal constructed by a third party (www.randomization.com).

#### Statistical Methodology

The intention-to-treat the population, which included all randomized patients to whom at least one dose of tolvaptan was administered, will be analyzed for the primary and secondary endpoints. As an exploratory analysis, we will evaluate interactions between tolvaptan add-on treatment and pre-specified subgroups on primary and secondary outcomes. Subgroups were defined for age (age > 65 and ≤ 65), eGFR at admission (eGFR 15–29 and eGFR 30–60), sodium and chloride levels at admission (above or below median), furosemide-naive or not, diuretic response achieved in the first 2 h after initial intravenous injection, blood urea nitrogen/creatinine (above or below median), and left ventricular ejection fraction ≤ 40 or >40%. A *P*-value of < 0.05 was considered statistically significant. Continuous variables are expressed as mean ± standard deviation if normally distributed or as median (interquartile range) if otherwise. When necessary, the variables were transformed for further analysis. Statistical significance was defined as two-sided 5%, and all analyses were performed using IBM SPSS^®^ (version 24.0) and R version 3.5.2 (R Foundation for Statistical Computing, Vienna, Austria; ISBN 3-900051-07-0, URL http://www.R-project.org). Normality was assessed using the Shapiro-Wilk test. The independent Student's *t*-test or Mann-Whitney U test was used to compare the two treatment groups according to each parameter. Categorical data will be displayed as a percentage or number and compared using Fisher's exact test. The relationship between baseline characteristics and outcomes will be compared by one-way analysis of variance, Kruskal-Wallis test, or χ^2^ test, as appropriate.

## Discussion

Despite their widespread use in the congestive heart failure, the evidence for intravenous loop diuretic administration is not as robust as many other treatments for heart failure (2). Loop diuretic agents work in a steep dose-response manner, in which dose intensification is often necessary to achieve a threshold concentration (ceiling) in invoking natriuresis (5). In AHF, this natriuretic threshold is increased, and the dose-response curves are shifted rightward in patients with concomitant kidney dysfunction (5), of which AHF accounts for 10 to 40% (16, 17). Although it has been established that a higher dose and a timely adjustment in the early stage of congestion should be implemented, there is still a need to determine the optimal dosing and timing strategy to overcome this diuretic resistance. The DOSE trial (3) demonstrated that a high loop diuretic strategy resulted in better decongestion (dyspnea improvement, weight change, and net fluid loss). However, this favorable effect was not investigated in the kidney dysfunction subgroup. Many trials have addressed the use of diuretics in patients with AHF and with kidney dysfunction. When the CARRESS-HF (18) trial compared ultrafiltration with a stepped approach to loop diuretic therapy in AHF patients with WRF, within 12 weeks before or 10 days after admission for heart failure and persistent congestion, renal failure developed in 15 and 18% of the stepped loop diuretic and ultrafiltration groups, respectively. This was despite the latter therapy being superior for preserving renal function 96 h after enrollment. However, WRF in AHF is a multifactorial condition, and an increase in serum creatinine does not always indicate a diuretic resistance. Evidence for appropriate diuretic therapy in patients with AHF with renal dysfunction and with diuretic resistance is still lacking. In 2019, the Heart Failure Association of the ESC suggested an aggressive diuretic treatment protocol (5). However, this has not been validated in AHF patients with renal dysfunction and with diuretic resistance.

Combination therapy offers many benefits in overcoming diuretic resistance. Most experts believe that it should not be initiated until the loop diuretic dose is up-titrated. However, no consensus has been reached regarding the time point for starting the add-on therapy. Although the combination diuretic treatment has recently gained more attention due to its physiologically synergistic action, its advantages may be outweighed by the substantial risk of electrolyte disturbances and a more severe WRF (2). Tolvaptan is promising for diuretic combination therapy. In the AQUAMARINE study (10), patients with AHF with renal dysfunction (defined as eGFR at admission 15–60 ml/min/1.73 m^2^) were randomized to tolvaptan add-on therapy or conventional diuretic treatment in the early stage of congestion (within 6 h after admission). This resulted in more diuresis and in better symptom relief without increasing the risk of WRF and electrolyte abnormalities. Recently, in a 3T trial (19), tolvaptan produced a higher cumulative urine output at 48 h and a lower risk of hyponatremia than the metolazone add-on therapy in patients with AHF and intravenous loop diuretics. The previous randomized trials and the current trial are summarized in Appendix 4. Overall, the DR-AHF trial is the first to evaluate the additive clinical value of treatment with tolvaptan in modern loop diuretic up-titrating therapy among patients with AHF and with verified diuretic resistance.

The DR-AHF trial has some limitations. First, as this is a single-center trial, some biases, such as selection bias, may not be entirely excluded. Moreover, this trial may be undersized to evaluate the prognostic impact of tolvaptan. Finally, this is an open-label trial in which the treatment of a doctor or the behavior of a patient can change.

In summary, the DR-AHF trial will demonstrate the efficacy of the timely intense loop diuretic monotherapy and tolvaptan combination treatment in the early stage of congestive heart failure with renal dysfunction and provide the clinical evidence of diuretic resistance.

## Ethics Statement

The studies involving human participants were reviewed and approved by the Independent Ethics Committee and Institutional Review Board at Nhan Dan Gia Dinh Hospital. Written informed consent was obtained from all participants for their participation in this study.

## Author Contributions

NGM, HNH, DM, and YM contributed equally to the design and implementation of this research. All authors have read and approved the manuscript.

## Funding

Otsuka Pharmaceutical Co. supports the study by providing the study drug and funding to the principal investigator. The funder was not involved in the study design, collection, analysis, interpretation of data, the writing of this article or the decision to submit it for publication.

## Conflict of Interest

YM is affiliated with a department endowed by Philips Respironics, ResMed, Teijin Home Healthcare, and Fukuda Denshi, and received an honorarium from Otsuka Pharmaceutical Co. The remaining authors declare that the research was conducted in the absence of any commercial or financial relationships that could be construed as a potential conflict of interest.

## Publisher's Note

All claims expressed in this article are solely those of the authors and do not necessarily represent those of their affiliated organizations, or those of the publisher, the editors and the reviewers. Any product that may be evaluated in this article, or claim that may be made by its manufacturer, is not guaranteed or endorsed by the publisher.

## Abbreviations

AHF: acute heart failure
ALT: alanine aminotransferase
AST: aspartate aminotransferase
AQUAMARINE: answering questions on tolvaptan's efficacy in patients with acute decompensated heart failure and renal failure
eGFR: estimated glomerular filtration rate
ESC: European Society of Cardiology
EVEREST: Efficacy of Vasopressin Antagonism in heart failure
NT-proBNP: N-terminal pro B-type natriuretic peptide
WRF: worsening renal function
TACTICS-HF: Targeting Acute Congestion with Tolvaptan in Congestive Heart Failure.
