# Supplementary material for: Tolvaptan Add-on Therapy to Overcome Loop Diuretic Resistance in Acute Heart Failure With Renal Dysfunction (DR-AHF): Design and Rationale
Source: Front Cardiovasc Med. 2022 Jan 27;8:783181. doi: 10.3389/fcvm.2021.783181 (PMC8829876; doi:10.3389/fcvm.2021.783181)
Supplement: Supplementary file 1 [file Data_Sheet_1.PDF]

Supplementary Material

Appendix 1. The DR-AHF trial assessment timings and study flow

At admission, AHF patients with wet-warm phenotype will be given a starting dose of intravenous furosemide which is 1-2 times of 24-hour oral furosemide home dose or 20mg – 40mg for loop diuretics naive. Patients will be asked to empty urinary bladder right before the first dose of intravenous furosemide. If they are suitable for study participation by meeting all inclusion and not violating any exclusion criteria, at 2 hour after the first intravenous furosemide, they will be randomly assigned 1:1 to receive a standard care with intravenous furosemide alone or a combination therapy with tolvaptan 15mg once daily for 2 days. In the both group, loop diuretic strategy will follow strictly the modified 2019 Position Statement from the ESC Heart Failure Association (*Figure 1*). It is not allowed to increase/decrease dose of tolvaptan during the 48h of this study unless adverse event is observed. After this period, the attending physicians will decide whether to continue tolvaptan therapy or not. The standard treaments of heart failure, including ACE-i, ARB, vasodilators, beta-blockers or digoxin, as well as the change from intravenous to oral loop diuretic or loop diuretic withdrawal are left to the attending physicians after 48 hours of admission.

Urine is collected and monitored carefully at 6 hours apart after randomization. Cumulative urine volume is calculated every 24 hours. Vital signs and symptom improvement assessed by 7-point Likert scale are recorded at admission, 24 hour and 48 hour after randomization. Serum sodium, potasium, chloride are evaluated at at admission, 12 hour, 24 hour and 48 hour, while urinary creatinine and sodium are sampled at randomization and 6 hours later. Serum urea and creatinine are re-assessed at 24 hour and 48 hour after randomization. In addition, aspartate aminotransferase (AST), alanine aminotransferase (ALT) and NT-proBNP are evaluated at admission and at the end of the study. A

summary of study assessment timings is available in *Appendix* 1. Echocardiography is performed by senior cardiologists at admission, 24 hour and 48 hour thereafter.

**Trial measurements**	Screening	Randomization	6h	12h	18h	24h	30h	36h	42h	48h
Demographics	X									
Past history	X									
Current treatment	X		X	X	X	X	X	X	X	X
Vital signs	X		X	X	X	X	X	X	X	X
NYHA class	X					X				X
Congestion phenotype	X									
Serum urea	X					X				X
Serum creatinine	X		X			X				X
Serum electrolyte	X			X		X				X
AST/ALT	X									X
NT-proBNP	X									X

Urine creatinine, chloride, pottasium and sodium		X	X			X				X
Echocardiography	X					X				X
Cumulative urine output		X	X	X	X	X	X	X	X	X
Cumulative i.v furosemide dose		X	X	X	X	X	X	X	X	X
Cumulative furosemide oral dose						X				X
Likert score		X				X				X
Body weight		X								X

Appendix 2. The DR-AHF trial echocardiography protocol

Echocardiography is performed by senior cardiologists at admission, 24 hour and 48 hour thereafter according to the echocardiography protocol described in *Appendix* 2.

**Variable**	**Acquisition**	**Analysis**
Peak E-wave velocity (cm/sec)	1. Apical four-chamber with color flow imaging for optimal alignment of PW Doppler with blood flow.<br>2. PW Doppler sample volume (1–3 mm axial size) between mitral leaflet tips.<br>3. Use low wall filter setting(100–200MHz) and low signal gain.<br>4. Optimal spectral waveforms should not display spikes or feathering.	Peak modal velocity in early diastole (after ECG T wave) at the leading edge of spectral waveform
Pulsed-wave TDI e' velocity (cm/sec)	1. Apical four-chamber view: PW Doppler sample volume (usually 5–10 mm axial size) at lateral and septal basal regions so average e' velocity can be computed.<br>2. Use ultrasound system presets for wall filter and lowest signal gain.<br>3. Optimal spectral waveforms should be sharp and not display signal spikes, feathering or ghosting.	Peak modal velocity in early diastole at the leading edge of spectral waveform
Mitral E/e'	See above for acquisition of E and e' velocity	MV E velocity devided by mitral annular e' velocity
TR systolic jet velocity (CW Doppler)	1. Parasternal and apical four-chamber view with color flow imaging to obtain highest Doppler velocity aligned with CW<br>2. Adjust gain and contrast to display complete spectral envelope without signal spikes or feathering	Peak modal velocity during systole at leading edge of spectral waveform
Left atrial maximal volume index (mL/BSA)	1. Apical four-and two-chamber: acquire freeze frame 1-2 frame before mitral valve opening	Method of area-length

	2. Left atrial volume should be measured in dedicated views in which left atrial length and transverse diameter are maximized	
Inferior vena cava size and collapsibility	1. Subcostal view: angling and rotating the transducer to the left from the subcostal four-chamber view.<br>2. M-mode: measurements of size changes throughout the respiratory cycle	

Appendix 3. The DR-AHF trial endpoints

Primary endpoint

Cumulative urine volume output at 48h after randomization

Secondary endpoint

1. Cumulative dose of furosemide at 48h after randomization
2. Symptom of dyspnea by 7-point Likert scale at 24h and 48h after randomization
3. Changes in body weight at 24h and 48h after randomization
4. Changes in serum urea, creatinine level and incidence of clinically relevant worsening renal function (WRF) at 24h and 48h after randomization
5. Changes in average e', E/e', LA volume, tricuspid regurgitation maximal velocity, IVC maximal diameter and IVC collapsibility 24h and 48h after randomization
6. Changes in serum sodium, potassium, chloride measured at 12h, 24h and 48h after randomization
7. Urine sodium, potassium and cloride excretion adjusted for urine creatinine at baseline, 6h and 24h after randomization
8. Changes in NT-proBNP at 48h after randomization and at discharge

Appendix 4. Comparison of previous randomized trials and DR-AHF

Trial	EVEREST	AQUAMARINE	TACTICS-HF	T3	SECRET of CHF	DR-AHF
**Design**	Randomized, double-blind, placebo-controlled study	Randomized, open-label study	Randomized, double-blind, placebo-controlled study	Randomized, double-blinded, double-dummy study	Randomized, double-blind, placebo-controlled study	Randomized, open-label study
**Sample size**	4133	217	257	60	250	Target 128
**Population**	Hospitalized with HF	Hospitalized with AHF	Hospitalized with AHF	Hospitalized with AHF	Hospitalized with AHF	Hospitalized with AHF
**Key inclusion criteria**	LVEF<=40%	Renal dysfunction (eGFR 15–60 mL/min/1.73m²)	BNP>400 or NT-proBNP>2000 pg/mL	Diuretic resistance	Diuretic resistance, renal dysfunction (eGFR<60 mL/min/1.73m²), or Na≤134 mEq/L	Diuretic resistance
**Definition of diuretic resistance**	NA	NA	NA	Total urine output of<2 L 12 h before enrollment while receiving IV loop therapy at a furosemide equivalent dosage of ≥240 mg/day over at least the previous 12 h	Urine output <= 125 mL/h during any ≥2-h period during the initial 8 h after IV administration of furosemide of at least 40 mg	Urine volume output < 300 mL within 2 h after the first dose of IV furosemide

**Comparison**	Conventional therapy + TLV vs. Conventional therapy + placebo	Conventional therapy + TLV vs. Conventional therapy only	Fixed-dose IV furosemide + TLV vs. Fixed-dose IV furosemide + placebo	Fixed-dose IV furosemide + TLV vs. Fixed-dose IV furosemide + oral metolazone vs. Fixed-dose IV furosemide + IV chlorothiazide	Conventional therapy + TLV vs. Conventional therapy + placebo	Fixed-dose IV furosemide + TLV vs. Fixed-dose IV furosemide only
**TLV dose and period**	30 mg, min 60 days	15 mg, 2 days	30 mg, 48 h	30 mg, 48 h	30 mg, max 7 days	15 mg, 2 days
**Fixed IV furosemide strategy**	Not determined	Not determined	According to DOSE trial (low dose arm)	According to CARRESS-HF trial	Not determined	According to HFA-ESC
**Primary outcome**	1. All-cause death 2. Cardiovascular death or hospitalization for heart failure	Urine volume at 48 h	The proportion of patients considered responders at 24 h	Weight loss at 48 h	Self-assessed 7-point dyspnea score at 8 and 16 h	Urine volume at 48 h

AHF, acute heart failure; eGFR, estimated glomerular filtration rate; IV, intravenous; LVEF, left ventricular ejection fraction; TLV, tolvaptan
